# Improvement in inpatient discharge planning for patients with alcohol use disorder with the implementation of a team-based multidisciplinary workflow

**DOI:** 10.1371/journal.pone.0306066

**Published:** 2024-06-25

**Authors:** Parinaz Abiri, Il Seok Daniel Jeong, Amber Verdell, Shivani Shah, Rana Entabi, Hoanglong Nguyen

**Affiliations:** 1 Department of Medicine, Olive View-UCLA Medical Center, Sylmar, CA, United States of America; 2 Department of Pharmacy, Olive View-UCLA Medical Center, Sylmar, CA, United States of America; 3 David Geffen School of Medicine, University of California, Los Angeles, Los Angeles, CA, United States of America; National University of Sciences and Technology, PAKISTAN

## Abstract

**Background:**

Alcohol use disorder (AUD) is a major economic and healthcare burden in the United States. While there is evidence-based medication-assisted treatment (MAT) for AUD, few physicians implement these therapies on a regular basis.

**Objective:**

To determine the impact of a pharmacy-guided AUD discharge planning workflow on the rate of MAT prescriptions and inpatient readmissions.

**Methods:**

This was a single-centered pre-and-post intervention study over a 6-month period, with a 90-day pre-intervention period and a 90-day post-intervention period. The study included all patients over the age of 18 years admitted to a medicine or surgery floor bed who presented with alcohol withdrawal at any point during their hospital course. The intervention involved a pharmacy workflow, in which a list of patients admitted with alcohol withdrawal was automatically generated and referred to pharmacists, who then provided recommendations to the primary physician regarding prescriptions for naltrexone, acamprosate, and/or gabapentin. The patients were then contacted within 30 days after discharge for post-hospitalization follow-up. Our outcome measures were change in prescription rate of MATs, change in total and alcohol-related 90-day readmission rates, and change in total and alcohol-related 90-day emergency department (ED) visit rates.

**Results:**

The pre-intervention period consisted of 49 patients and the post-intervention period consisted of 41 patients. Our workflow demonstrated a 195% increase in the prescription rate of MATs at discharge (p < 0.001), 61% reduction in 90-day total readmission rate (p < 0.05), 40% reduction in 90-day total ED visit rate (p = 0.09), 92% reduction in 90-day alcohol-related readmission rate (p < 0.05), and 88% reduction in 90-day alcohol-related ED visit rate (p < 0.05).

**Conclusions:**

Our intervention demonstrated that a pharmacy-based AUD discharge planning workflow has the potential to reduce inpatient readmissions and ED visits for patients with AUD, thus demonstrating improved patient outcomes with the potential to reduce healthcare costs.

## Introduction

Alcohol use disorder (AUD) affects roughly 28.6 million adults in the United States (US) [1], consisting of about 15% of all young adults (age 18 to 25) and 11% of all older adults (age over 25). Excessive and dangerous alcohol consumption poses a significant financial burden to the US economy, with the total annual economic cost of AUD estimated to be $249 billion, of which $27 billion has been attributed to healthcare costs alone [2]. AUD is graded on a spectrum of mild, moderate, to severe, with increased severity of the disorder having a higher likelihood of resulting in life-threatening medical complications, such as encephalopathy, seizures, coma, and death. In fact, the average annual number of alcohol-attributable deaths in the US is estimated to be over 140,000, more than all illicit substances combined, making it the third-leading preventable cause of death in the US [3].

Therapies for AUD range from behavioral interventions to pharmacotherapies. Currently, there are 3 FDA-approved medication options: naltrexone (approved in 1994), acamprosate (approved in 2004), and disulfiram (approved in 1949) [4]. Multiple clinical trials studying these medications have demonstrated decreased number of drinking days, decreased rate of relapse, reduced cravings, and a lower rate of treatment withdrawal [5–11]. Gabapentin has also been used off-label with multiple recent studies revealing the high efficacy of gabapentin in the treatment of AUD [12]. However, while these medications offer outstanding efficacy and many patients report an interest in MAT, their real-life use has been limited, with only a shocking 0.9% of patients with AUD receiving medication-assisted treatment (MAT) in 2021 [1].

With an estimated 17% to 33% of admitted patients nationwide suffering from alcohol-related problems [13–15], the inpatient setting presents a ripe environment for capturing patients who might otherwise not be regularly seen and screened for AUD in an outpatient setting by a primary care provider. This environment thus demonstrates a notable opportunity gap to improve management of AUD. Prior studies have aimed to improve inpatient discharge prescription rate of Naltrexone through provider education [16, 17]. In the short-term, these interventions, as well as retrospective studies assessing the same, have demonstrated that improved MAT discharge rate reduces 30-day emergency department visits and admissions [16–18]. However, due to the challenges of maintaining training practices and compliance in a time-constrained environment, we aimed to develop a novel and durable inpatient workflow to maintain long-term improvement in prescription and readmission rates without the need for provider training.

We thus describe a single-center pre-and-post study using a pharmacy-based intervention with the aim to improve prescription of medication-assisted treatments for AUD and reduce inpatient readmission rates secondary to alcohol abuse.

## Methods

### Intervention design & development

**Fig 1:**
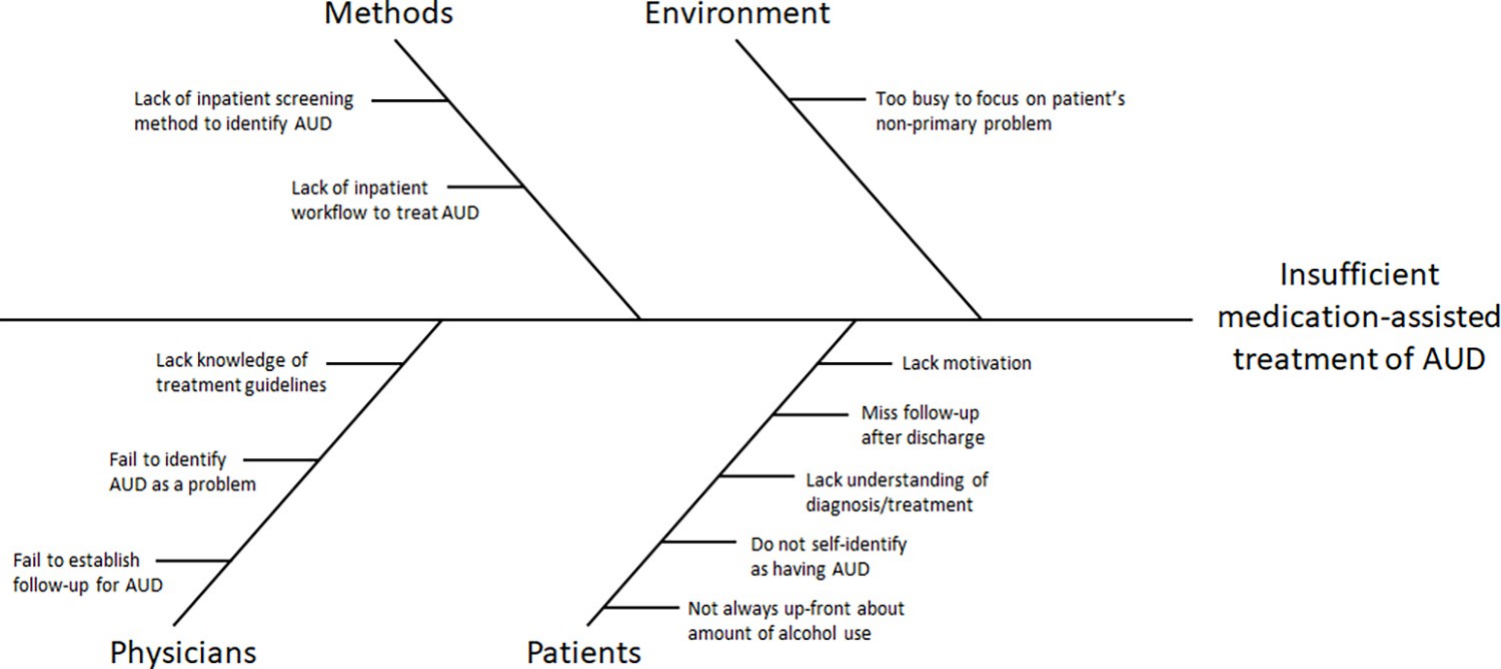
Root-cause analysis. By identifying the primary reasons for low prescription rate of MATs for AUD, we were able to target a key hospital resource to resolve several of the major failure points.

We performed a root-cause analysis to determine the primary reasons that patients were not being properly treated for their AUD in the inpatient setting (Fig 1). We then sought to identify the barriers to change and the stakeholders that could most effectively impact such a change while remaining cost-effective and time-conscientious in the fast-paced inpatient environment. As such, we identified inpatient pharmacists as primary stakeholders who physicians often trust as a resource in medical decision making. We thus based our intervention around our pharmacist’s interaction with the patient’s primary team.

In our implementation, patients were screened based on the activation of an order set in the Electronic Health Records (EHR) written for alcohol withdrawal based on the Clinical Institute Withdrawal Assessment (CIWA) protocol. This selection criterion is based on a likely high concordance rate between patients presenting with alcohol withdrawal and those with moderate-to-severe alcohol use disorder per Diagnostic and Statistical Manual of Mental Disorders (DSM–5) criteria [19]. The presence of at least two symptoms from the DSM-5 criteria indicate AUD and patients presenting with alcohol withdrawal often qualify for several of these criteria, thereby acting as an effective screening mechanism. Admission or discharge diagnoses of the patients were independent of their concurrent diagnosis for AUD.

**Fig 2:**
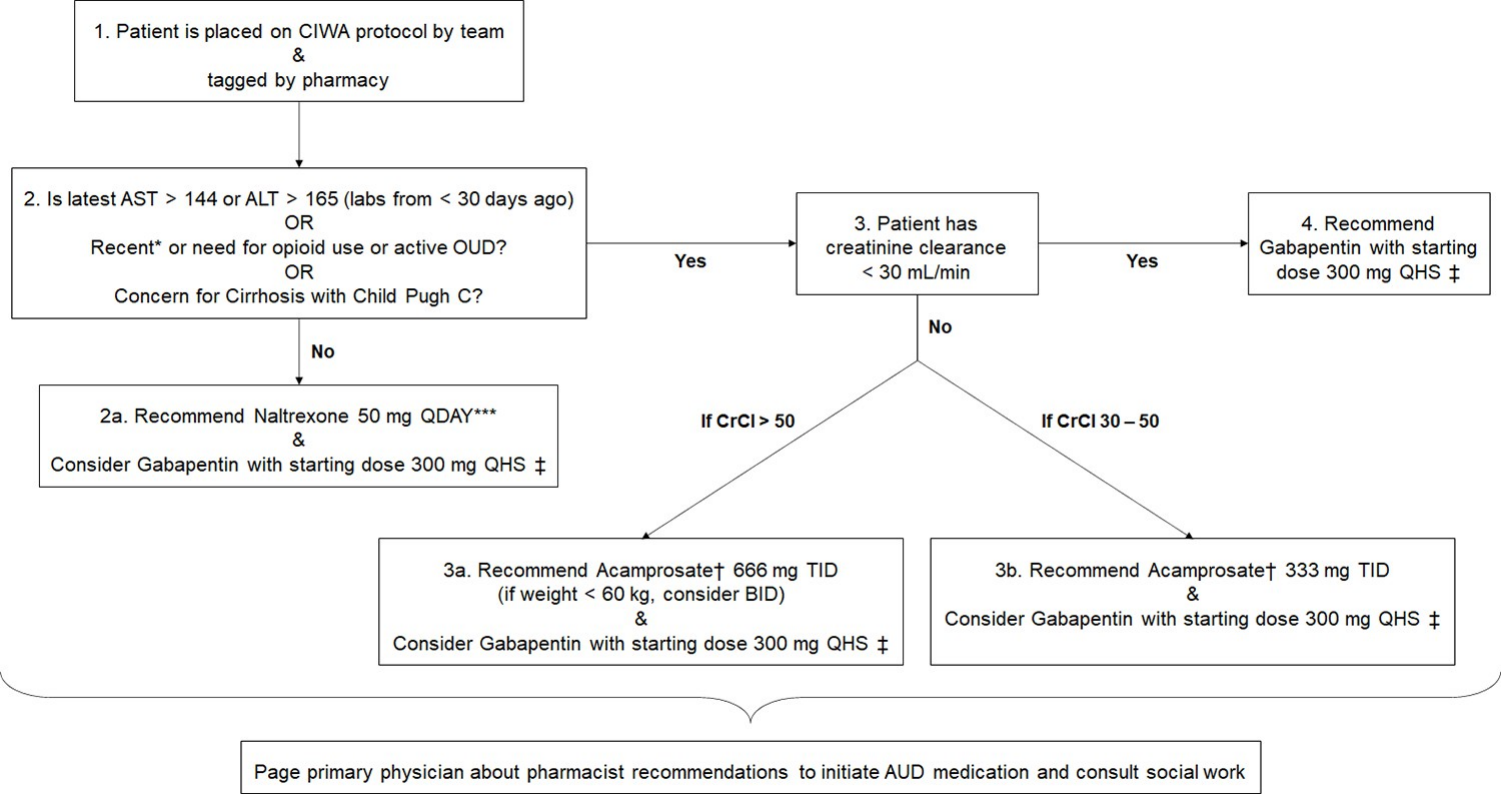
Pharmacy-based workflow for MAT of AUD. We designed an evidence-based workflow to guide the process followed by inpatient pharmacy. A pharmacist would follow the pathway above to identify the appropriate management. They then notified a medicine team member of their recommendations for AUD medications prior to patient discharge. *Recent is defined as short-acting opioids in the past 7 days, extended-release opioids in the past 10 days, and long-acting opioids in the past 14 days. **Inform the primary team that if there are concerns for side effects, can start at 25 mg QDAY for 3 days, then titrate to 50 mg QDAY. ‡Can recommend titration goals to the primary physician with caution for excess sedation. †Recommend to start medication once detoxification/withdrawal is done. CIWA: Clinical Institute Withdrawal Assessment; AST: aspartate transaminase; ALT: alanine transaminase; OUD: opioid use disorder; QDAY: daily; QHS: nightly; TID: three times daily; BID two time daily; AUD: alcohol use disorder; CrCl: creatinine clearance.

In our workflow, inpatient pharmacy automatically tagged all patients on whom the CIWA protocol order set was activated by the inpatient medicine team. Trained pharmacist then identified the recommended MAT for qualifying patients based on their medical history and latest guidelines. These medications included naltrexone, acamprosate, and gabapentin; disulfiram was excluded from the protocol due to the wide range of contraindications, extensive education needed, and variable follow-up in patients being discharged from an inpatient setting. The recommendations were then paged out to the primary medicine team prior to discharge to ultimately identify the diagnosis of AUD and prescribe medications as they saw appropriate. Only the pharmacy team was aware of the ongoing study; the primary team’s sole point of interaction with the study was through the pharmacist via a unidirectional page with an optional call back number for questions regarding the medication recommendation. The workflow as described is shown in Fig 2.

We further implemented a post-discharge follow-up phase to ensure continued medication compliance. During our root-cause analysis and planning phase, we observed that many patients who were being discharged with MAT were in fact unaware of its indication, thereby impacting their likelihood of compliance. We established a collaboration with the substance use disorder bridge clinic to ensure a phone follow-up within 30 days following hospital discharge to assess the patients’ perception of their new AUD medication as well as their status of medication adherence and their changed behavior in regards to alcohol use. This would also further allow for patients who initially refused medication on discharge to have additional counseling a period after recovering from their acute hospitalization. Phone contact information was documented in all patients on admission to ensure the possibility of follow-up on discharge; if patients disclose lack of phone access, our social workers are able to provide them with phones.

### Study design

We performed a pre-and-post study analysis over a period of 6 months, with a 90-day pre-intervention period (PRE) from June 1, 2022 to August 29, 2022 and a 90-day post-intervention period (POST) from September 8, 2022 to December 6, 2022. In POST, the workflow of Fig 2 was implemented during a patient’s admission period after which outcome measures were calculated. The study was performed at Olive View-UCLA Medical Center (OVMC), a 377-bed, university-affiliated county hospital primarily serving the medically indigent and uninsured population of the suburban area of the Greater Los Angeles Area. Our quality improvement (QI) study was evaluated by the OV-UCLA Institutional Review Board (IRB) and accepted for IRB exemption. Patient records were accessed on April 15, 2023 for research purposes. The authors had access to patient information during the initial retrospective data collection; the requirement for informed consent was waived for this study.

Inclusion criteria included all patients over the age of 18 years admitted to a medicine floor bed who had activation of the aforementioned order set at any point during their hospital course. Exclusion criteria included any patient who was already on a MAT (naltrexone, acamprosate, and/or gabapentin) *and* who was discharged on the same medication without a new prescription, as this would indicate no direct intervention in AUD management. Of note, these medications were tagged as MATs for AUD after manual chart review only if prescribed for the purpose of treating AUD, and not any other indications, such as neuropathy or opioid use disorder.

Our outcomes were measured by reviewing patient data across Los Angeles (LA) hospitals by directly reviewing documentation identifying ICD-10 diagnoses in Cerner ORCHID EHR for LA County hospitals, including Olive View-UCLA Medical Center, Los Angeles General Medical Center, and Harbor-UCLA Medical Center. Our primary outcomes were change in prescription rate of MATs, change in total 90-day readmission rates, and change in total 90-day emergency department (ED) visit rates. Our secondary outcomes included change in 90-day alcohol-related (diagnoses covered by ICD-10 code F10) readmission rates and change in 90-day alcohol-related ED visit rates. Baseline characteristics of our patients are listed in Table 1.

**Table 1:** Patient baseline characteristics.

Characteristics	Pre-Intervention (N = 49)	Post-Intervention (N = 41)	P-value
Age (mean +/- SD)	48 +/- 11	42 +/- 11	0.007
Sex no. (%)			0.119
Male	38 (77%)	36 (88%)	
Female	12 (24%)	5 (12%)	
Ethnic Group no. (%)			0.376
Hispanic or Latino	33 (66%)	30 (73%)	
Not Hispanic or Latino	17 (34%)	11 (27%)	
Elevated LFTs no. (%)			0.159
Yes	11 (22%)	4 (10%)	
No	39 (78%)	37 (90%)	
Cirrhosis no. (%)			0.800
Yes	12 (24%)	8 (20%)	
No	38 (76%)	33 (81%)	
OUD no. (%)			1.00
Yes	2 (4%)	1 (2%)	
No	48 (96%)	40 (98%)	
Chronic Pain no. (%)			1.00
Yes	1 (2%)	0 (0.00%)	
No	49 (98%)	41 (100%)	
DHS Empanelment no. (%)			0.288
Yes	23 (46%)	14 (34%)	
No	27 (54%)	27 (66%)	
Insurance no. (%)			0.522
Medicare & Commercial	8 (16%)	5 (12%)	
Medi-Cal	31 (62%)	30 (73%)	
Medi-Cal Restricted & Self-Pay	11 (22%)	6 (15%)	

Definitions: Elevated LFTs (Liver Function Tests) at the time of discharge: defined by AST > 144 or ALT > 165. Cirrhosis: defined by imaging (Computed Tomography or Ultrasound) and laboratory findings (blood counts, coagulation studies, and liver function panel) suggestive of cirrhosis. OUD: defined by active opioid use disorder. Chronic pain: defined by pain requiring regular use of opioid medications for pain relief. DHS (Department of Health Services) empanelment: defined by the presence of assigned primary care physician within the Los Angeles County health system.

### Data & statistics

Data analysis for 90-day readmission rates and ED visit rates (inclusive of those that led to an admission) were computed based on the ratio of the number of occurrences to the number of patients:

Readmission rate = total number of readmissions / total number of patients

ED visit rate = total number of ED visits / total number of patients

Statistical analyses were performed using R (version 4.2.2; The R Foundation for Statistical Computing) in RStudio (version 2022.12.0). A p-value of < 0.05 was considered statistically significant. Fisher exact tests and unpaired t-tests were conducted to evaluate differences in categorical and continuous variables with two groups, respectively. Chi square test was used to evaluate categorical variables with more than two groups.

There was no significant difference between our PRE and POST groups in most baseline characteristics. However, a significant difference was found between the patient’s ages in the two groups (p-value = 0.005). To ensure lack of confounding factors, logistic regression was conducted to assess for associations between age and outcomes. There was no significant association found between age and prescription rate (p-value = 0.501), age and readmission rate (p-value = 0.449), age and ED visit rate (p-value = 0.275), age and alcohol-related readmission rate (p-value = 0.494), or age and alcohol-related ED visit rate (p-value = 0.937).

### Balancing measure

The cost of our intervention relates to the amount of time spent by the pharmacy team to obtain training on the workflow in Fig 2, analyze the patient chart per the algorithm, follow through with paging the patient’s primary team, and respond to any of their follow-up questions. A post-intervention questionnaire with the following questions was thus released to the pharmacy staff to assess for this cost:

1. How satisfied are you with the current inpatient workflow for recommending medications to Medicine teams for patients diagnosed with AUD? (1 is least satisfied, 5 is very satisfied)
2. How much time do you estimate the current AUD inpatient pharmacy workflow costs you personally in a week?
3. Any additional comments on workflow obstacles, response rate, frustrations, or things you liked/didn’t like?

## Results

The total number of patients admitted to OVMC that met the inclusion criteria was 49 in the 90-day PRE group and 43 in the 90-day POST group. From the 43 patients in the POST group, 1 patient was excluded as he was not discharged within the timeline of the study due to placement issues and 1 patient was excluded as an error in the paging process resulted in the pharmacy recommendation page not to be sent out to the primary team, thus resulting in a total of 41 patients in the POST group.

**Fig 3:**
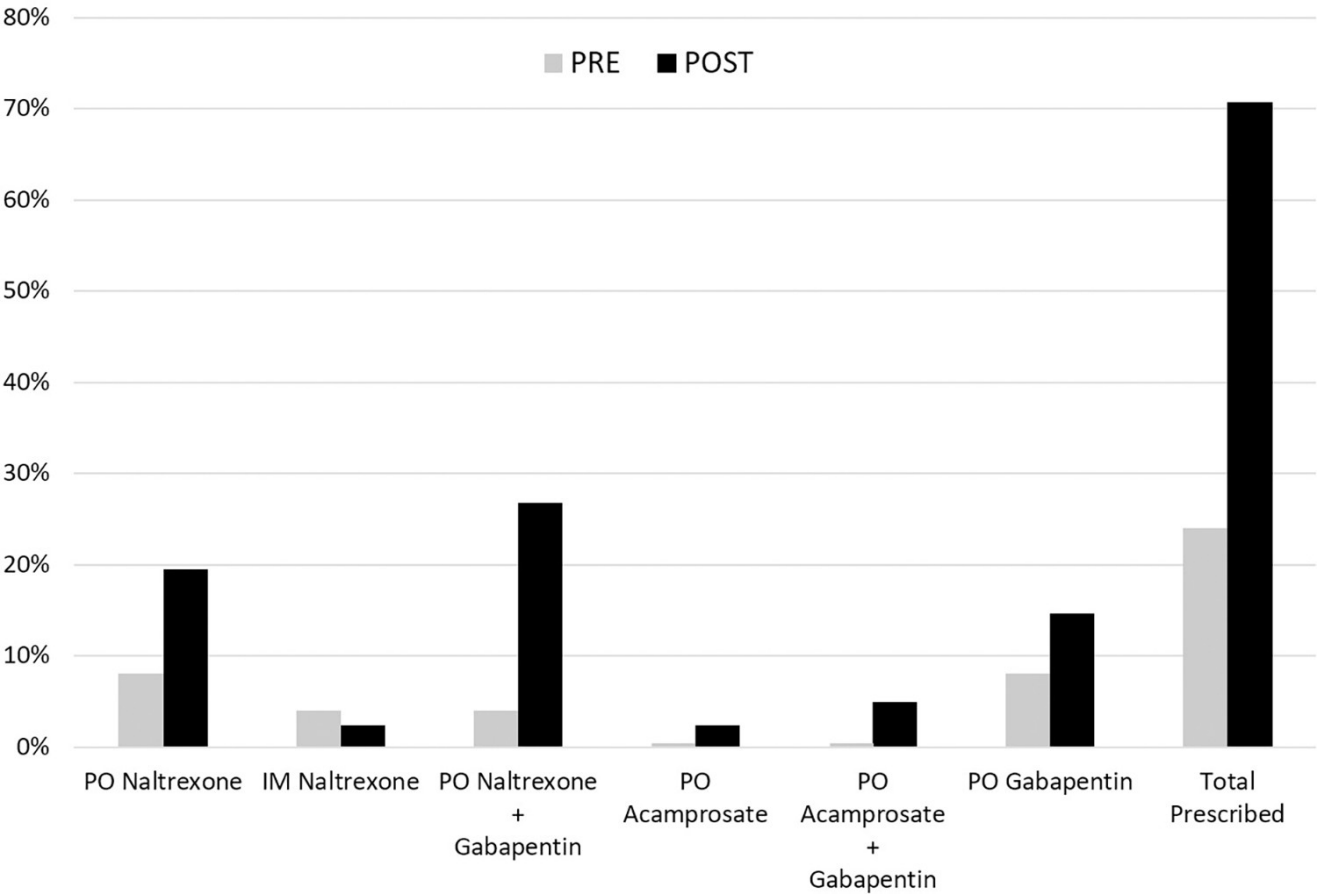
MAT prescription rates. Rates of individual medications prescribed at discharge as well as total MATs prescribed significantly improved with our pharmacy-led intervention. ** for p-value < 0.001.

From the included patients, 24% were discharged with a new prescription for AUD medications in the PRE group, while 71% were discharged with a new prescription for AUD medications in the POST group, demonstrating a 195% improvement in discharge prescription rate using the pharmacy-driven protocol (Table 2 & Fig 3). These medications included oral or intramuscular (IM) naltrexone, oral acamprosate, and oral gabapentin. The initial IM naltrexone dose was injected prior to discharge.

**Table 2:** Pre- and post-intervention MAT prescription rates.

	PO NX	IM NX	PO NX + GA	PO AC	PO AC + GA	PO GA	TP
**PRE no. (%) **	4 (8%)	2 (4%)	2 (4%)	0 (0%)	0 (0%)	4 (8%)	12 (24%)
**POST no. (%) **	8 (20%)	1 (2%)	11 (27%)	1 (2%)	2 (5%)	6 (15%)	29 (71%)

PO: oral; IM: intramuscular; NX: naltrexone; GA: gabapentin; AC: acamprosate; TP: total prescribed

To evaluate the impact of appropriate AUD medication management, we monitored 90-day hospital readmissions as well as total ED presentations to Los Angeles hospitals. Among patients in the PRE group, total 90-day readmission rate was 0.44, in comparison to patients in the POST group with readmissions rates of 0.17, demonstrating a significant 61% reduction in total readmissions (p < 0.05). Notably, historical 90-day readmission data from all patients discharged from OVMC was 22.10% in fiscal year 2021–2022 [20]. Our study demonstrated that once the impact of AUD was minimized, patients’ readmission rate was reduced to nearly that of the hospital average based on their typical chronic medical conditions.

In comparing ED visits among the two groups, while we also observed a rate reduction of 40%, from 0.77 in the PRE group to 0.46 in the POST group, this difference was not found to be statistically significant (p = 0.09). For our secondary outcomes, we found a significant reduction of 92% in alcohol-related readmission rates from 0.29 in the PRE group to 0.02 in the POST group (p < 0.05). In this category, we also found a significant rate reduction of 88% in ED visits from 0.40 in the PRE group to 0.05 in the POST group (p < 0.05).

Post-discharge outpatient follow-ups were also monitored as part of the newly established AUD discharge planning workflow in the POST group. Among the 41 patients in this group, post-discharge outreach within a 30-day period was successful in 19 patients (46% outreach rate). Among these 19 patients, 1 patient was excluded due to transition to hospice, 1 patient was excluded as adherence was not documented in the post-visit summary, and 1 patient was excluded as his health deteriorated and his AUD medications were contraindicated pending improvement in clinical course. From the remaining 16 patients, 10 patients (63% success rate) had remained adherent with their AUD medications, 3 patients (19%) reported sobriety using non-MAT techniques (e.g. Alcoholic Anonymous), and 1 patient who was never initiated on an AUD medication continued to refuse treatment.

Finally, we assessed the impact of our intervention on pharmacy workflow. Based on our survey results, the satisfaction rating for our intervention was an average of 4.67 (out of 5). The amount of time spent ranged from 1 to 3 hours per week per pharmacist; however, due to pharmacist rotation schedules, multiple pharmacists may have been assigned to the intervention in a given week, each with a variable time assignment to the protocol. On average, the inpatient unit supervisor reported an estimated 30 minutes per day spent total on the intervention.

## Discussion

Over 800,000 years of potential life lost can be attributed to excessive alcohol use alone [3], not accounting for the damage to the patients’ quality of life as well as that of those around them. A value-assessment of AUD has found that healthcare costs constitute the second most major cost secondary to AUD, with the remainder attributed to losses in workplace productivity, law enforcement dues, and losses from motor vehicle accidents. Of these costs, 40% are paid by federal, state, and local governments, thus impacting society as whole [2]. We thus aimed to improve clinical treatment of AUD by utilizing an existing resource in our hospital system, the pharmacy department. Our novel pharmacy-based intervention for the inpatient discharge prescription of AUD MATs demonstrated a near two-fold increase in the prescription rate of these medications. We further demonstrated a meaningful decrease in 90-day readmissions and ED revisitations. Inpatient pharmacists are a reliable source of information, guidance, and auditing in most hospitals, thus allowing a seamless integration into our existing patient care workflow.

In our root-cause analysis, we demonstrated four global causes of insufficient medication-assisted treatment of AUD; with our workflow incorporating the inpatient pharmacy team, we were successfully able to address these barriers: (1) a knowledge gap on AUD treatment guidelines creates a hesitancy in prescribing the appropriate therapies; this was addressed by having recommendations delivered by pharmacists who are well-versed in medications indications and contraindications and who were provided with a pre-defined evidence-based workflow (Fig 2) for reference; (2) the time-constraints of the primary physician prevents focus on non-clinically urgent problems such as long-term management of AUD; loss of time can be avoided by getting pre-determined recommendations from a pharmacist; (3) there is a lack of screening for patients with AUD who could benefit from MAT; this was addressed by having the pharmacy department identify patients placed on the CIWA protocol through their automated system; (4) lastly, the knowledge gap of patients reduced their medication adherence; this was addressed with inpatient initiation of medications where the patient may otherwise be unaware of such potential therapies, as well as post-discharge follow-up and management as available for those with successful outreach.

There are also positive *long-term* implications to this workflow. Despite the additional time resource required by inpatient pharmacy, the improvement in patient care and reduction in readmission rates can reduce healthcare costs. Whereas prior studies utilized one-time training sessions to motivate MAT prescription [16–18], this implementation provides a durable solution, without time-dependent loss of efficacy due to knowledge leak or lapsed importance. In fact, the recurrent reminders in paged out recommendations to the physician can ultimately serve as a self-training exercise, creating an environment where the process becomes a learned behavior.

There were some notable limitations to our study. This was a single-centered study, with narrow patient demographics, and a fairly small sample size of 40 to 50 patients per sample group. Given AUD is a chronic disease, our findings over an extended period of follow-up with a larger sample size may provide additional insight into patient behavior as well as the apparent correlation between increased medication prescription and reduced acute care utilization. Furthermore, we may consider scheduling our PRE and POST group into the same time period during the year to examine the effect of our intervention during times in which new trainees, faculty, and staff are recruited as well as the impact of holidays and seasonal changes. For example, our PRE study period with low rate of MAT prescription rates coincided with the beginning of the academic year and the start of new trainees. The low rate of treating AUD echoes the neglect this disease receives, especially when there are time constraints and knowledge variation. These time periods may benefit even more from and further highlight the need for our proposed pharmacy-led workflow. We further aim to optimize our post-discharge follow-up workflow to increase outreach success rate. To assess the impact on variable patient populations, we would also like to implement this process at other hospital sites, where integration into the existing pharmacy-provider relation may require process attenuation. In our next cycle, we also aim to expand this process beyond patients on CIWA protocol, seeking out a method to identify patients without alcohol withdrawal but with moderate-to-severe AUD.

## Conclusion

The findings in this study suggest that pharmacy-based discharge planning for alcohol use disorder can lead to improved patient outcomes by increasing MAT prescription rate as well as decreasing 90-day readmission rates. Although the study was only conducted over a 90-day period for each group, the results suggest a remarkable improvement in clinical outcomes. In addition, a survey of our pharmacy team suggests that this intervention does not significantly increase work load or decrease work satisfaction of inpatient pharmacists, indicating that this model is sustainable for long-term use. With future studies aimed at providing further improvement and expansion of the presented workflow, this model for discharge planning can hopefully be applied on a larger scale to improve outcomes for the many patients with AUD, all while reducing healthcare costs.

## References

[1] Richesson D, Magas I, Brown S, Hoenig JM. Key Substance Use and Mental Health Indicators in the United States: Results from the 2021 National Survey on Drug Use and Health. Rockville, MD; 2022 Dec.

[2] Sacks JJ, Gonzales KR, Bouchery EE, Tomedi LE, Brewer RD. 2010 National and State Costs of Excessive Alcohol Consumption. Am J Prev Med. 2015 Nov 1;49(5):e73–9. doi: 10.1016/j.amepre.2015.05.031 26477807

[3] Centers for Disease Control and Prevention [Internet]. [cited 2023 Feb 19]. Alcohol Related Disease Impact (ARDI) Application. Available from: https://nccd.cdc.gov/DPH_ARDI.

[4] Fairbanks J, Umbreit A, Kolla BP, Karpyak VM, Schneekloth TD, Loukianova LL, et al. Evidence-Based Pharmacotherapies for Alcohol Use Disorder: Clinical Pearls. Mayo Clin Proc. 2020 Sep 1;95(9):1964–77. 32446635 10.1016/j.mayocp.2020.01.030

[5] Kirchoff RW, Mohammed NM, McHugh J, Markota M, Kingsley T, Leung J, et al. Naltrexone Initiation in the Inpatient Setting for Alcohol Use Disorder: A Systematic Review of Clinical Outcomes. Mayo Clin Proc Innov Qual Outcomes. 2021 Apr;5(2):495–501. 33997645 10.1016/j.mayocpiqo.2021.01.013 PMC8105524

[6] Reus VI, Fochtmann LJ, Bukstein O, Eyler AE, Hilty DM, Horvitz-Lennon M, et al. The American psychiatric association practice guideline for the pharmacological treatment of patients with alcohol use disorder. Vol. 175, American Journal of Psychiatry. American Psychiatric Association; 2018. p. 86–90. doi: 10.1176/appi.ajp.2017.1750101 29301420

[7] Anton RF. Naltrexone for the Management of Alcohol Dependence. New England Journal of Medicine. 2008 Aug 14;359(7):715–21. 18703474 10.1056/NEJMct0801733 PMC2565602

[8] Susanne R, Andrea HH, Stefan L, hert Philippe L, Simona V, Michael S. Acamprosate for alcohol dependence. Cochrane Database of Systematic Reviews. 2010 May 28;128(6):379.

[9] Jonas DE, Amick HR, Feltner C, Bobashev G, Thomas K, Wines R, et al. Pharmacotherapy for adults with alcohol use disorders in outpatient settings: A systematic review and meta-analysis. JAMA. 2014 May 14;311(18):1889–900. 24825644 10.1001/jama.2014.3628

[10] Srisurapanont M, Jarusuraisin N. Opioid antagonists for alcohol dependence (Review). Cochrane Database of Systematic Reviews [Internet]. 2008;(3). Available from: http://www.thecochranelibrary.com. 10.1002/14651858.CD001867 10908513

[11] Yahn SL, Watterson LR, Olive MF. Safety and Efficacy of Acamprosate for the Treatment of Alcohol Dependence. Subst Abuse. 2013 Jan 1;7. 10.4137/SART.S9345 PMC3565569 23399877

[12] Anton RF O’malley SS, Ciraulo DA, Cisler RA, Couper D, Donovan DM, et al. Combined Pharmacotherapies and Behavioral Interventions for Alcohol Dependence The COMBINE Study: A Randomized Controlled Trial. JAMA [Internet]. 2006;295(17):2003–17. Available from: www.jama.com. 16670409 10.1001/jama.295.17.2003

[13] Doering-Silveira J, Fidalgo TM, Silva Nascimento CLE, Alves JB, Seito CL, Saita MC, et al. Assessing alcohol dependence in hospitalized patients. Int J Environ Res Public Health. 2014 May 28;11(6):5783–91. 24879488 10.3390/ijerph110605783 PMC4078547

[14] Suen LW, Makam AN, Snyder HR, Repplinger D, Kushel MB, Martin M, et al. National Prevalence of Alcohol and Other Substance Use Disorders Among Emergency Department Visits and Hospitalizations: NHAMCS 2014–2018. J Gen Intern Med. 2022 Aug 1;37(10):2420–8. 34518978 10.1007/s11606-021-07069-w PMC8436853

[15] Roche AM, Freeman T, Skinner N. From data to evidence, to action: Findings from a systematic review of hospital screening studies for high risk alcohol consumption. Drug Alcohol Depend. 2006 Jun 9;83(1):1–14. 16310323 10.1016/j.drugalcdep.2005.10.011

[16] Wei J, Defries T, Lozada M, Young N, Huen W, Tulsky J. An Inpatient Treatment and Discharge Planning Protocol for Alcohol Dependence: Efficacy in Reducing 30-Day Readmissions and Emergency Department Visits. J Gen Intern Med. 2015 Mar 1;30(3):365–70. 25092008 10.1007/s11606-014-2968-9 PMC4351284

[17] Stephens JR, Moore C, Stepanek K V, Garbutt JC, Starke B, Liles A, et al. Implementation of a Process for Initiating Naltrexone in Patients Hospitalized for Alcohol Detoxification or Withdrawal. J Hosp Med [Internet]. 2018;13(4):221–8. Available from: https://shmpublications.onlinelibrary.wiley.com/doi/abs/10.12788/jhm.2900. 29364992 10.12788/jhm.2900

[18] Calabrese J, Brown J, Hasan M, Neuhut S, Jo Y. Hospital Readmission in Alcohol Use Disorder Patients: The Role of Anti-Craving Medications and Discharge Disposition. HCA healthcare journal of medicine. 2022;3(2):39–45. 37426377 10.36518/2689-0216.1243 PMC10324689

[19] Diagnostic and statistical manual of mental disorders. 5th ed. American Psychiatric Association; 2013.

[20] Annual Report to the Los Angeles County Board of Supervisors Fiscal Year 2021–22. Sylmar, CA.

